# Use of single photon metal artefact reduction in the detection of an impacted capsule endoscope

**DOI:** 10.1259/bjrcr.20170050

**Published:** 2017-08-04

**Authors:** Kevin Z Zhou, Robert Khor, Kenneth K Lau

**Affiliations:** ^1^Department of Diagnostic Imaging, Monash Medical Centre, Monash Health, Melbourne, VIC, Australia; ^2^School of Clinical Sciences, Faculty of Medicine, Nursing and Health Sciences, Monash University, Clayton, VIC, Australia

## Abstract

Wireless capsule endoscopy was introduced over a decade ago and is now a widely used tool in the investigation of gastrointestinal pathologies. Despite its ubiquity, the full profile of indications, contraindications and complications is still being developed. Metal artefact reduction is a software technique which can significantly reduce the artefact produced by metallic objects on CT scans. This case exemplifies a rare but noteworthy complication of capsule endoscopy and highlights a novel application of metal artefact reduction.

## Case presentation

An 80-year-old male was being investigated at a tertiary hospital for iron deficiency anaemia. His medical history, symptomology and physical examination did not indicate a cause. After gastroscopy and colonoscopy were also unremarkable he was referred for capsule endoscopy. The delivery of the capsule in the morning was routine; however, review of the images 8 hlater showed a static picture and no evidence the capsule had entered the stomach. The patient was clinically stable and contacted to present to the emergency department with a suspicion of capsule impaction.

## Investigations

**Figure 1.**
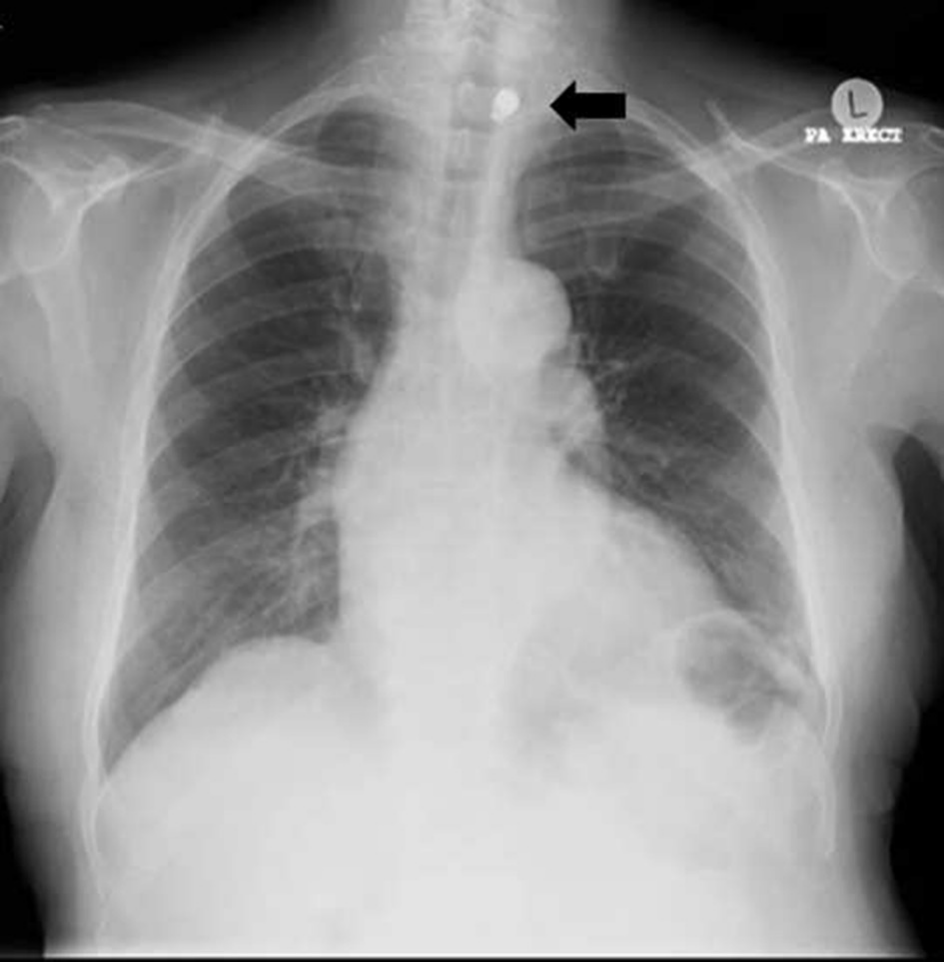
Plain chest radiograph displayed a metallic object (arrow) at the thoracic inlet to the left of midline at *T*_2_ level.

**Figure 2.**
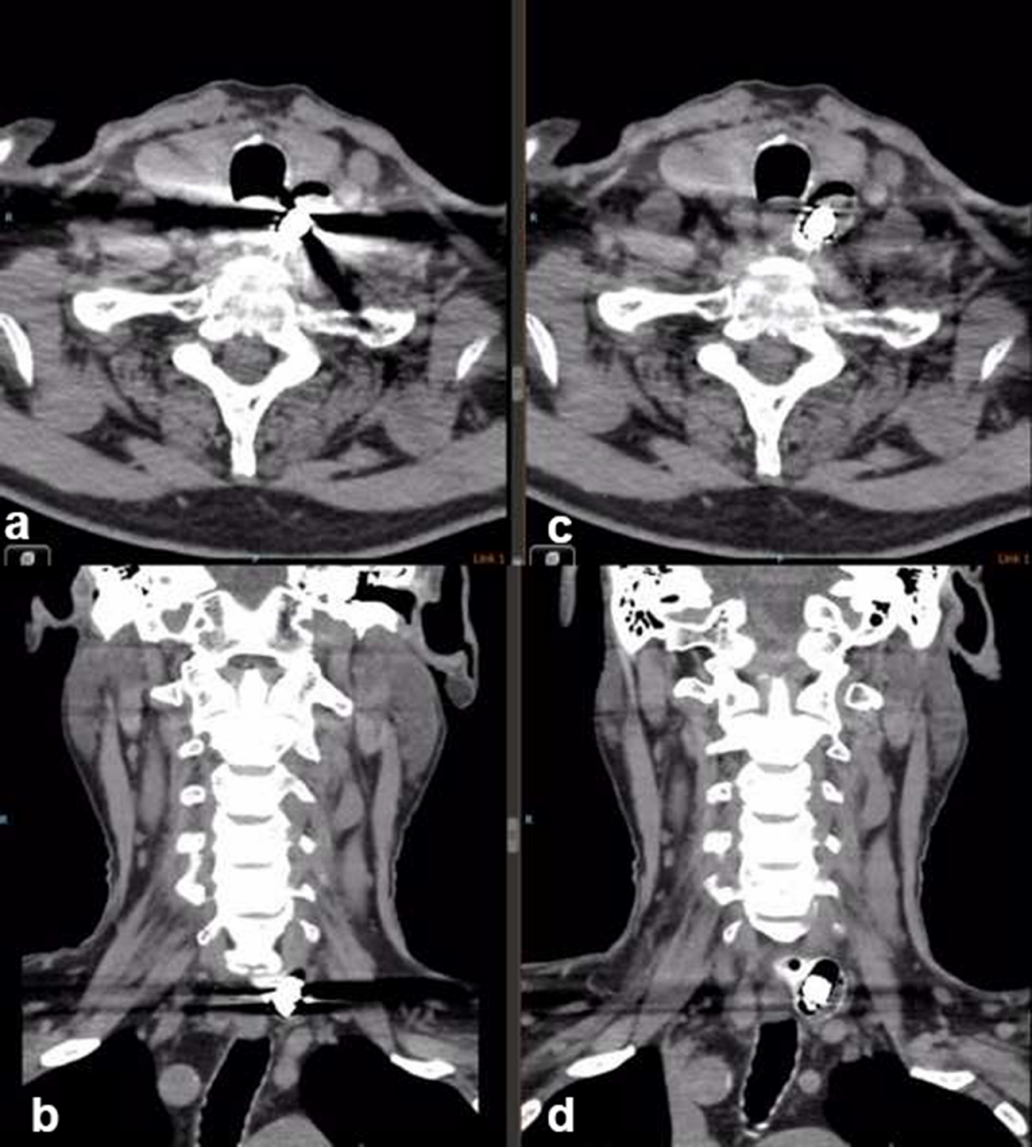
Non-contrast CT of neck in axial (a and c) and coronal (b and d) reformats showed the capsule endoscope at the left lower neck. The metal aliasing artefacts obscure the adjacent soft tissues (a and b). The endoscope in Zenker’s diverticulum was better depicted after the artefacts were markedly reduced with metal artefact reduction.

A chest radiograph demonstrated a metallic object at the level of the second thoracic vertebra to the left of the trachea (Figure 1). The patient had no known oesophageal or tracheal diverticulum and expressed no symptoms of dysphagia. To pinpoint the exact anatomical location of the foreign body a non-contrast CT scan of the neck (Figure 2) was performed. The effective dose of CT was 0.81 mSv. This confirmed the object was the capsule endoscope lying to the left of the hypopharynx. The adjacent soft tissue was obscured by aliasing metal artefacts arising from the capsule. A single photon metal artefact reduction (MAR) software technique (O-MAR; Philips Healthcare, Cleveland, OH) was applied which successfully removed most of the metallic artefacts. It revealed the capsule was impacted in an oesophageal diverticulum.

## Outcome and follow-up

The capsule was successfully endoscopically removed under a general anaesthetic and the patient’s recovery was uncomplicated. A follow-up barium swallow study confirmed the presence of a Zenker’s diverticulum which the patient opted not to surgically repair as he was asymptomatic. He was also offered endoscopic insertion of the capsule endoscope to bypass the diverticulum; however, the patient refused. His anaemia resolved with iron transfusions but no definitive cause was found. He was discharged from the clinic.

## Discussion

Capsule endoscopy has been proven over the past decade to be a safe and effective test with minimal complications and few contraindications.^1–3^ On review of the English-language literature only five case reports of capsule endoscope impaction in a Zenker’s diverticulum were found.^4–8^ Of those cases, four were diagnosed on plain radiography and contrast swallow while one proceeded immediately to endoscopy based on the live video feed. In all cases the impacted capsule was removed within 48 hwithout complication. This is the first reported case to use any form of cross-sectional imaging to aid in diagnosis and assessment.^4–8^

Metallic devices, because of their high density and atomic number, create significant aliasing artefact on a CT scan through a combination of beam hardening, scatter, edge effects and photon starvation.^9^ This manifests as white black streaks and some areas of complete information loss making accurate localization of the capsule endoscope difficult. O-MAR is an example of single-energy iterative MAR technique and is a software method which can markedly reduce metal artefact. MAR’s key feature is the subtraction of metal traces from the original image before it enters the iterative reconstruction loop. It can be applied retrospectively on the acquired CT dataset and does not require repeat CT scanning or an increased radiation dose.^9^ The additional reconstruction only takes a few minutes and does not create significant processing delays. It greatly improves image quality and diagnostic value, in particular of the soft tissues around the metallic object on CT, in a timely manner. The most common application of MAR is in cases of orthopaedic joint replacements and internal fixation devices which are often encountered on CT scans. Non-orthopaedic objects such as cerebral aneurysm clips, pacemakers and foreign bodies will cause the same level of image degradation and also benefit from MAR techniques.^9^ The use of MAR in this case allowed much clearer visualization of the diverticulum, aiding in confirming its exact location and excluding any complications arising from the impacted capsule such as perforation or haematoma.

The primary drawback of performing a CT scan is the increased radiation dose delivered to the patient. The benefits of accurate and complete knowledge of the pathology must be weighed against the risks of radiation exposure. Modern CT scanners are generally able to limit and reduce the radiation dose through the use of iterative reconstruction technique. The effective dose in our patient is sub-milli-Sievert. Active discussion with the treating team and the patient is integral to decide on the best course of investigation and management for each individual patient.

## Learning points

1. The case presents a rare but noteworthy complication of capsule endoscopy.
2. A metallic object adjacent to the hypopharynx in the lower neck and thoracic inlet should alert the reporting radiologist to the possibility of an impacted capsule endoscope or other foreign body.
3. A CT scan in these cases can provide valuable information, especially in cases where there are no pre-existing conditions or known pathologies. The use of CT should be weighed against the risk of radiation exposure.
4. Where aliasing artefact from metal objects is obscuring adjacent soft tissue, metal artefact reduction techniques can significantly improve image quality, add diagnostic confidence and aid in further assessment.

## Consent

Written informed consent was obtained from the patient for publication of this case report, including accompanying images.
